# Gastrointestinal Manifestations and Low-*FODMAP* Protocol in a Cohort of Fabry Disease Adult Patients

**DOI:** 10.3390/nu15030658

**Published:** 2023-01-28

**Authors:** Giorgia Gugelmo, Nicola Vitturi, Francesco Francini-Pesenti, Ilaria Fasan, Livia Lenzini, Romina Valentini, Gianni Carraro, Angelo Avogaro, Paolo Spinella

**Affiliations:** 1 Division of Clinical Nutrition, Department of Medicine (DIMED), University Hospital, University of Padova, 35122 Padova, Italy; 2 Division of Metabolic Diseases, Department of Medicine (DIMED), University Hospital, University of Padova, 35122 Padova, Italy; 3 Department of Medicine (DIMED), University Hospital, University of Padova, 35122 Padova, Italy; 4 Division of Nephrology, Department of Medicine (DIMED), University Hospital, University of Padova, 35122 Padova, Italy

**Keywords:** Fabry disease, gastrointestinal manifestations, low-*FODMAP* diet

## Abstract

Fabry disease (FD) is an X-linked lysosomal disorder caused by α-galactosidase A enzyme deficiency. Gastrointestinal (GI) manifestations are reported in FD with a prevalence of about 50%, usually treated by Enzymatic Replacement Therapy (ERT) or oral treatment. Since FODMAPs (*Fermentable Oligosaccharides, Disaccharides, Monosaccharides, and Polyols*) can be involved in GI manifestations and dysbiosis in FD patients, a low-*FODMAP* diet could represent an alternative adjunctive treatment in FD subjects, as well as being useful for reducing symptoms in Irritable Bowel Syndrome (IBS). We retrospectively assessed data from 36 adult FD patients followed at the Inherited Metabolic Rare Diseases Adult Centre of the University Hospital of Padova (mean age 47.6 ± 16.2 years). Patients were screened for GI symptoms by IBS severity score and Gastrointestinal Symptom Rating Scale (GSRS) questionnaires. In symptomatic patients, the low-*FODMAP* diet was proposed in order to improve GI manifestations; it consists of a phase of elimination of fermentable saccharides, succeeded by a gradual reintegration of the same. Severe or moderate GI symptoms were found in 61.1% of patients, with no correlation to the therapy in use, and significantly more severe in the classical form of FD. The protocol was completed by seven patients affected by severe GI manifestations, significantly higher than the others. The low-*FODMAP* diet significantly improved indigestion, diarrhoea, and constipation. This dietetic protocol seemed to have a positive impact on intestinal symptoms, by identifying and reducing the intake of the foods most related to the onset of disorders and improving the clinical manifestations. A low-*FODMAP* diet may be an effective alternative approach to improve intestinal manifestations and quality of life, and nutrition can play an important role in the multidisciplinary care of patients with FD.

## 1. Introduction

Fabry disease (FD) (Online Mendelian Inheritance in Man [OMIM] #301,500) is an X-linked lysosomal rare disorder caused by different mutations within the α-galactosidase A (AGAL/GLA) gene, resulting in AGAL enzyme deficiency.

FD’s worldwide incidence has been estimated at 1 in 40,000 to 1 in 117,000 live male births [1], but recent data from new-born screenings assessed a higher incidence of 1:3200 by including late onset or mild GLA variants [2].

AGAL enzyme deficiency causes lysosomal accumulation of glycolipids (globotriaosylceramide [Gb3]), and major damages are reported in the kidneys, heart, nervous system, and skin; progressive globotriaosylceramide [lyso-Gb3] accumulation can lead to a high risk of early onset of stroke, life-threatening arrhythmia, myocardial infarction, or cardiac and renal failures [3,4].

FD patients experience pain and nonspecific gastrointestinal (GI) symptoms that can also occur as a first manifestation [5]. Data from the Fabry Outcome Survey registry reported a prevalence of 51% of GI symptoms in FD patients [6].

The most common manifestations are abdominal pain and diarrhoea, followed by constipation, nausea, and vomiting [7]. These symptoms often appear in childhood and can be life-threatening, with a significant impact on quality of life [5,7,8].

Abdominal pain is the most frequent symptom, manifested by colic with pain in the mid or lower abdomen, bloating, cramping, or midabdominal discomfort [7,9]. These signs may increase during or after meals or be triggered by stress, but FD patients do not show significant differences in body mass index (BMI) due to a reduced food intake related to GI symptoms [7,10].

Two types of treatments are currently used for FD: the enzyme replacement therapy (ERT; every other week, intravenously), or the chaperone therapy (every other day, orally) [11,12], aimed at blocking the progress of the disease and also improve the bowel symptomatology; however, many FD patients still manifest GI symptoms [5].

Some drugs, including loperamide, metoclopramide, proton pump, and simethicone for flatulence and bloating seem to be useful in some GI manifestations [7,13,14]. Several therapies typically used to treat Irritable Bowel Syndrome (IBS) have been proposed, such as linaclotide against constipation or, with caution in cardiac unstable conditions, antispasmodic dicyclomine against spasms [7,15,16].

FD GI symptoms are indeed similar to Irritable Bowel Syndrome (IBS), where a low-*FODMAP* diet (Fermentable Oligosaccharides, Disaccharides, Monosaccharides, and Polyols) has proved effective [8,17,18,19,20]. Short-chain fermentable carbohydrates induce an osmotic effect by drawing water into the intestinal and/or colonic lumen, so FODMAPs in the distal ileum and colon can be fermented to short-chain fatty acids and gases, enhancing GI manifestations [18,19].

Since many FODMAPs require AGAL to be digested, FD patients appear potentially responsive to this dietary protocol [7,9,21]. It usually consists of two different phases: 4–6 weeks of elimination phase on a low-*FODMAP* diet, followed by a gradual reintroduction phase of FODMAP-rich foods in order to define which ones are tolerated or not [17,18]. Some IBS patients benefit from orally recombinant AGAL administration; in FD patients also, it can support nutrients’ digestion, improve pro-inflammatory status, and reduce dysbiosis by depleting intestinal lyso-Gb3 [7,22,23]. Particularly in FD patients, dysbiosis and overgrowth of intestinal flora can be due to reduced AGAL activity in the intestine, leading to improper galactooligosaccharide digestion [7,24].

As recommended for IBS patients, some dietary advice can help in improving GI manifestations, such as limiting alcohol, spicy foods, caffeine, lactose, dietary fibre, and also short-chain fermentable carbohydrates [19,20,25].

Since there is clinical efficacy of the low-*FODMAP* diet in patients with IBS [20,26], this dietary protocol can represent thereby an interesting nutritional approach in FD patients [17,21]. If on the one hand it requires adherence by patients, on the other hand it is a non-drug treatment that could be effective in improving GI symptoms and consequently, also the quality of life of FD patients [7,17].

Given that data from the literature are still lacking in FD adult patients treated with a low-*FODMAP* protocol, we performed a retrospective study to evaluate it in a cohort of FD adult patients. We assessed the prevalence of GI manifestations and then the effectiveness of a low-*FODMAP* protocol in patients with GI symptoms.

## 2. Materials and Methods

We retrospectively assessed data from adult patients affected by FD who were followed at the Inherited Metabolic Rare Disease Adult Centre of the University Hospital of Padova.

No exclusion criteria were applied. All FD patients presented genetic and enzymatic diagnosis.

The complete medical history and the physical assessment were recorded from each patient at the routine outpatient multidisciplinary visit.

At the first visit, we assessed anthropometrics by a mechanical scale with movable weights and an altimeter (Seca 700, weight precision: 50 g; height precision: 0.5 cm; seca gmbh & co kg, Hammer Steindamm 3-25, 22089 Hamburg, Germany) for the weight and height assessments, respectively. Body mass index (BMI) was obtained by the ratio between weight (kg) and height squared (m^2^), and it was interpreted according to the World Health Organisation (WHO) classification (BMI < 18.5 kg/m^2^ = underweight range, 18.5 < BMI < 25 kg/ m^2^ = healthy weight range, 25 < BMI < 30 kg/m^2^ = overweight range, BMI > 30 kg/m^2^ = obesity range).

In addition, general health was measured by a 12-item Short Form Survey (SF-12) [27] at the first evaluation, a test evaluating the two scores of physical health index (PSC12) and mental health index (MSC12).

At the first visit, GI symptoms were assessed by two validated tests: Gastrointestinal Symptom Rating Scale (GSRS) [28] and IBS severity score questionnaires [29].

GSRS is a questionnaire including 13 items depicting problems with satiety, abdominal pain, diarrhoea, constipation, and bloating.

IBS severity score is a validated test incorporating pain, distension, bowel dysfunction, and quality of life/global well-being. Patients were considered symptomatic when IBS severity score was >75 points, and GI manifestations were divided into severe (>175 points), moderate (175–75 points), or absent (<75 points).

GI symptomatic patients were recommended the low-*FODMAP* dietetic protocol, which consists of two different phases: 4–6 weeks of elimination of FODMAPs, and 8–10 weeks of gradual reintegration (Table 1). Phase 1 consists of the total exclusion of foods containing FODMAPs, which can be categorized into four main groups according to the fermentation bowel processes: Oligosaccharides: fructans and galactooligosaccharides (GOS)—fructans, also known as fructo-oligosaccharides (FOS), are chains of the sugar fructose of different lengths. The main dietary sources of these are wheat products (bread/breakfast cereal/pasta), some vegetables (e.g., onion, garlic, artichoke) and as an ingredient added to some processed foods as a prebiotic (e.g., FOS, oligofructose, or inulin). GOS are chains of the sugar galactose. Main dietary sources are pulses, beans, legumes, and cashew or pistachio nuts; Disaccharides: lactose, present in dairy products such as milk, soft cheese, and yogurt; Monosaccharides: fructose, found in fruit, honey, corn syrups (widely used in the food industry); Polyols: sugar alcohols such as sorbitol, mannitol, isomalt, maltitol, xylitol, which occur naturally in some fruits and vegetables, but are also used as artificial sweeteners in sugar-free chewing gum, mints, and other low-calorie or sugar-free products.

At the end of the FODMAP elimination phase, we evaluated the effects on intestinal symptoms by GSRS and monitored anthropometrics.

Phase 2, meanwhile, consists of a gradual reintroduction of foods containing FODMAPs by consuming small quantities of one food from a different main FODMAP group every two or three days. It was suggested to patients to record a diary of FODMAP foods reintroduced to monitor the possible effects on bowel complaints.

**Figure 1:**
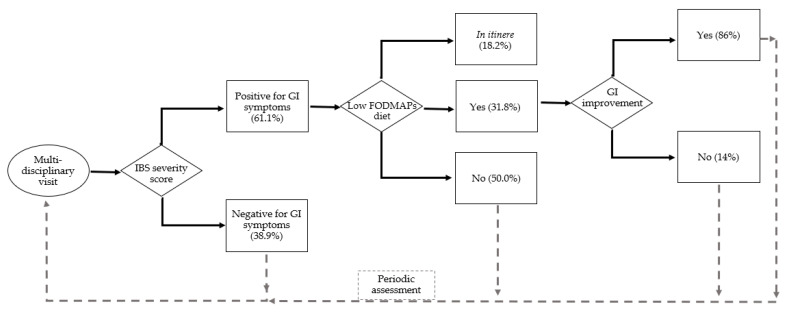
The flowchart of FD patients: first evaluations at the multidisciplinary visit (n. 36) and assessment for GI manifestations by IBS severity score (positive = n. 22), patients that accepted the low-*FODMAP* protocol (n. 7 completed and n. 4 *in itinere*).

In Figure 1, we reported a flowchart representing our FD patients’ treatment path, from the assessment for GI manifestations at the first multidisciplinary visit to the proposal of the low-*FODMAP* protocol in relation to the IBS severity score positive for bowel complaints, until possible improvement of the score. Non-symptomatic patients (both adhering or not to the FODMAP protocol) were addressed at their periodic multidisciplinary assessment at our Inherited Metabolic Rare Disease Adult Centre.

Data analyses were performed using Microsoft^®^ Excel 2019 (Microsoft Italia, Viale Pasubio 21, 20154 Milan, Italy) and GraphPad Prism 9 (GraphPad Software, Franklin Street 225, 02110 Boston, MA, USA). A descriptive statistical study of the sample was completed by using the parameters of centralization (mean and median) and dispersion (standard deviation (SD), maximum, and minimum), according to a variable type.

T-tests were used to compare means of different subgroups, and Pearson’s test was used to establish correlations (*p* value < 0.05, confidence interval 0.95).

## 3. Results

We recorded data from 36 adult patients with FD.

### 3.1. Subject Characteristics

The general characteristics of the subjects are summarized in Table 2 and, more in detail, in Supplementary Materials, Table S1.

All individuals were Caucasian, n. 36 adult patients with mean age of 47.6 ± 16.2 years (17–82 years); 36.1% were males, 63.9% were females.

Genetic mutations were all involved in the GLA gene; 25% were associated to the classical form and 63.9 % to the late onset form, and 11.2% were variants of uncertain significance (VUS) type.

The distribution of FD therapy at the first evaluation was: 44.5% enzymatic treatment, 19.4% oral treatment, 36.1% none. Probiotics and prebiotics were not prescribed to any patient.

The SF-12 test showed a mean physical health index (PSC12) of 45.6 ± 7.2 vs. mean Italian population value of 48.6 and a mean mental health index (MSC12) of 51.5 ± 11.2 vs. a mean Italian population value of 49.9.

Mean BMI was 25.6 ± 6.0 kg/m^2^ (overweight range).

### 3.2. Gastrointestinal Manifestations

GI symptoms were found in n. 22 patients (61.1%), by the IBS severity score (Table 3 and more in detail in Supplementary Materials, Figure S1), of whom 33.3% presented severe manifestations and 27.7% referred moderate symptoms.

Mean IBS severity score was 116.2 ± 97.8, indicative of moderate bowel manifestations.

In the GI symptomatic patients (n. 22), 27.3% were males and 72.7% were females; no significant correlation was found between sex and the severity of GI manifestations.

Of the same group, 41% (n. 22 GI symptomatic patients) presented a classical form, 50% a late onset form, and 9% a VUS. IBS severity score was higher in patients with classical form than late onset ones (*p* < 0.01); 45.5% were on ERT, 22.7% received oral therapy, and 31.8% received none.

In GI symptomatic patients (n. 22), mean BMI was 24.6 ± 5.7 kg/m^2^ (healthy weight range), not significantly different from non-symptomatic subjects (n. 14).

Moreover, in the total population of n. 36 FD patients, no difference was observed in BMI or in IBS severity score among the three groups of therapy (ERT vs. oral vs. none).

**Figure 2:**
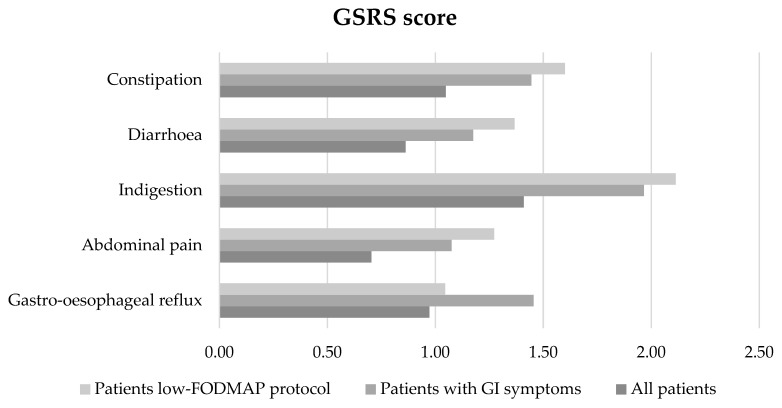
Characteristics of GI manifestations by GSRS (constipation, diarrhoea, indigestion, abdominal pain, gastro-oesophageal reflux) in all subjects (n. 36), in patients with IBS severity score positive for GI symptoms (n. 22), and in patients adhering to the low-*FODMAP* diet (n. 11).

In all subjects (n. 36), the most frequent GI manifestations detected by GSRS were: indigestion, constipation, gastro-oesophageal reflux, diarrhoea, and abdominal pain. In the patients treated with the low-*FODMAP* protocol, the most frequent symptoms were: indigestion, constipation, diarrhoea, abdominal pain, and gastro-oesophageal reflux (Figure 2).

Among the total population (n. 36 subjects), a positive significant correlation between IBS severity score and the GI manifestations by GSRS were found: gastro-oesophageal reflux (*p* < 0.01, r = 0.61), abdominal pain (*p* < 0.01, r = 0.45), indigestion (*p* < 0.01, r = 0.56), and constipation (*p* < 0.01, r = 0.63), but not diarrhoea.

SF12 score in GI symptomatic patients (n. 22) was: PCM12 = 45.5 ± 6.8; MCM12 = 49.8 ± 12.6; no significative correlation was noted between the severity of GI manifestations and physical and mental health in this group of patients (n. 22) and among all patients (n. 36).

### 3.3. Low-*FODMAP* Diet

The low-*FODMAP* protocol was suggested to all n. 22 patients with GI manifestations and IBS severity score positive (>75).

N. 7 FD patients started and completed the protocol; n. 4 patients started the low-*FODMAP* protocol (phase 1) but did not complete it in time to evaluate the improvement of GI manifestations. N. 11 patients (50% of symptomatic patients) did not start the low-*FODMAP* protocol for personal reasons. Furthermore, 63.6% of this group was on FD therapy and they preferred to treat GI symptoms only with oral or ERT treatment.

The patients adhering to the low-*FODMAP* protocol (n. 11) were: 63.7% females, 27.3% males; 54.5% classical form, 36.4% late form, and 9% VUS; 54.5% were on ERT, 27.3% were on oral treatment, and 18.2% were not on therapy; SF12 score: PCM12 = 46.8 ± 8 and MCM12 = 48.7 ± 11.7, both not significantly different from all the samples. Mean BMI in patients adhering to a low-*FODMAP* protocol (n. 11) was 23.1 ± 5.3 kg/m^2^ (healthy weight range).

Mean IBS severity score in this subgroup was 195.2 ± 63.5 (indicative of severe GI manifestations), and was significantly higher than the score of patients who did not follow the low-*FODMAP* protocol (n. 25, *p* < 0.01). In addition, indigestion and constipation detected by GSRS were higher than in the other patients (*p* < 0.05).

The low-*FODMAP* diet was found to be effective in reducing GI symptoms in 6/7 (86%) patients who completed the protocol, and only 1 VUS female did not improve her GI symptoms during the Phase 1.

**Figure 3:**
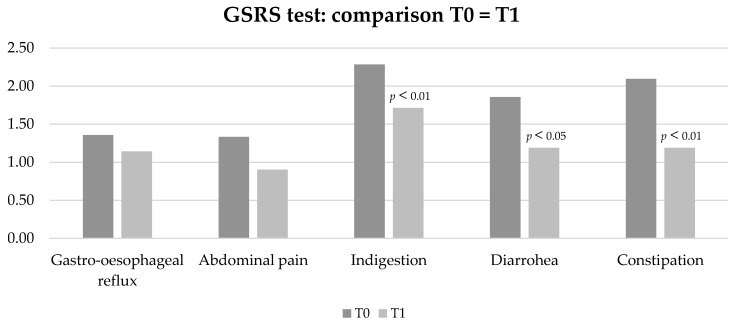
Characteristics of GI manifestations by GSRS (constipation, diarrhoea, indigestion, abdominal pain, gastro-oesophageal reflux) in patients on low-*FODMAP* diet before (T0) and after Phase 1 of the protocol (T1).

In particular, comparing GSRS at T0 (time of first evaluation) and T1 (at the end of Phase 1), the low-*FODMAP* protocol improved the manifestations of indigestion (*p* < 0.01), diarrhoea (*p* < 0.05), and constipation (*p* < 0.01) (Figure 3). Patients completing the dietetic protocol presented a mean BMI = 21.7 kg/m^2^ and weight was not significantly different at the end of low-*FODMAP* phase 1 and phase 2.

Foods that patients indicated as more involved in GI symptoms were those rich in gluten and lactose, and some vegetables mainly of *Brassicacae* and *Lyliaceae* species.

## 4. Discussion

Our study is the first to evaluate the efficacy of a low-*FODMAP* diet in adult patients with FD, and also accurately assess the prevalence and the characteristics of GI manifestations.

FD adults represent a growing population, thanks both to progress in genetics and medicine that allowed the detection of new disease variants, and also to the screening of special risk populations (e.g., patients on dialytic treatment or renal transplanted) [30,31,32]. Moreover, drugs such as ERT or oral therapy have helped to extend life expectancy and implement the future number of FD patients [31]. Up to now, many studies investigated GI manifestations in FD [5,7,9,33,34], but the efficacy of a low-*FODMAP* protocol in GI symptom treatment has not been evaluated until now.

The literature reported that diarrhoea occurs in 20% of FD patients with a frequency up to 12 times a day, and it is more frequent in males (26%) than in females (17%) [6,35]. Nausea and vomiting are more common in children, while constipation mainly affects females [7,12]. In FD adult patients, other GI manifestations are gastritis, haemorrhoids, chronic intestinal pseudo-obstruction, diverticula disease, and bowel ischemia [5,7].

Our FD patients revealed a prevalence of GI symptoms higher than the data from the Fabry Outcome Survey registry [6] (61.1% vs. 51%), with a mean IBS severity score indicative of manifestations of moderate intensity. The main reported symptoms were indigestion, constipation, and gastro-oesophageal reflux, and the significant correlation between the IBS severity score and the specific manifestations by GSRS for gastro-oesophageal reflux, abdominal pain, indigestion, and constipation reflects the severity of these manifestations in our FD patients.

GI symptoms in classical form patients were found to be significantly higher than in late onset ones, independently of the prescribed therapy. ERT or oral treatment seem to have a heterogeneous effect on the improvement of GI manifestations, because GI symptoms are present also in patients on therapy and no correlation between severity of GI manifestations and the prescribed therapy was found in our patients. Moreover, in our FD symptomatic patients, physical health was actually lower than the mean Italian population (45,8 vs 48,6), reflecting the negative impact on their quality of life from the GI manifestations [5] without regard to FD target treatment (ERT or oral therapy).

As reported by other authors [7], no evidence of malnutrition was found in patients with GI symptoms.

FD has a conspicuous economic impact because of the cost of expensive therapies, such as ERT and chaperone therapy (~250,000 €/year per patient), in particular if GI signs are the only observed manifestations in affected patients [7]. Adjunctive treatments are often used to treat GI symptoms in FD patients, including metoclopramide and H-2 blockers for delayed gastric emptying, and dyspepsia and other pharmacotherapy for the treatment of dysmotility and diarrhoea [11].

Patients who decided to start a low-*FODMAP* protocol showed at first more severe symptoms than those who did not follow the protocol, probably affecting their quality of life and motivating them to an alternative treatment such as a dietetic protocol.

The low-*FODMAP* diet was positively useful in patients followed by our centre, since it significantly helped in improving their bowel manifestations and it represented an alternative functional treatment in symptomatic subjects without side effects.

This dietetic protocol also allowed patients to identify and minimize the intake of the foods most related to the clinical GI signs (gluten, lactose, and some vegetables).

However, a low-*FODMAP* diet is not easy to practice, and some patients can not adhere to this treatment or can drop out *in itinere*. In our sample, 50% of subjects with moderate or severe manifestations did not start the low-*FODMAP* diet despite their symptomatology. Patient adherence is basic for the success of any dietetic therapy and only a few studies have examined patient adherence to a low-*FODMAP* diet and its acceptability [36]. Some reasons could be that this protocol in its initial period (Phase 1) can be difficult to follow and a little expensive [36].

The efficacy of a low-*FODMAP* diet in our patients suggests that in FD patients, the lack of AGAL potentially reduces digestion within the gut of a FODMAP-rich diet. Moreover, globotriaosylsphingosine (lyso-Gb3) could promote dysbiosis, and a low-*FODMAP* diet can be helpful in improving intestinal gut health, since FODMAPs can increase bowel water content, production of gas, and excessive production of short-chain fatty acids (SCFAs) [18,36].

A strength of this study is that the data belong to a single Inherited Metabolic Rare Diseases Adult centre with a multidisciplinary team specifically dedicated to FD adult patients. An important limitation is the small number of analysed patients due to the low prevalence of FD adult patients, and the non-adherence to the dietetic protocol by several subjects. Long-term outcomes in patients adhering to a low-*FODMAP* protocol were not assessed and more prolonged follow-up is necessary.

Another limitation is represented by the fact that currently there are no guidelines for assessing GI manifestations in FD patients [5]. In our study, we used validated tests, but further studies and more agreement for the screening of GI manifestations in FD patients are needed. Considering the relevant presence of bowel symptoms in these subjects, more attention and consideration should be given also to other possible causes of abdominal complaints (e.g., allergy IgG-dependent or food intolerance).

## 5. Conclusions

Given the high prevalence of GI signs that affect the quality of life and the heterogeneous efficacy of ERT or oral therapy on these manifestations, in FD symptomatic patients a low-*FODMAP* protocol can be a useful option to treat GI symptoms, which is less expensive and can improve their quality of life. Further studies and research into the dietary treatment of these patients should be encouraged. Nutrition can play an important role and nutritionists and/or dietitians expert in the field should take part in the multidisciplinary care of these rare patients.

## Figures and Tables

**Table 1:** Low-*FODMAP* protocol proposed to our FD GI symptomatic patients, and then retrospectively analysed in this study.

Food Group	Foods to Be Restricted	Foods Allowed
Dairy products	Cow’s, sheep’s, buffalo’s, and goat’s milk, soft cheeses, cream (sour and whipped), yogurt, mozzarella with lactose, béchamel cottage cheese and cheese sauces, milk chocolate.	Lactose-free cow’s milk, butter, aged cheese (e.g., Parmesan), lactose-free yogurt, lactose-free cheese
Dairy alternatives	Coconut milk and cream, soy milk	Almond milk, kefir, rice milk
Cereals	Bread, pasta, breadsticks, pastry products, … if made with wheat (wheat), various flours (chickpea flour, lentil flour, pea flour, soy flour), spelt, rye, barley, kamut, cous-cous, semolina	Breadsticks, pasta and pastry products (products with wheat-free cereals and flours: quinoa, rice, oats, buckwheat, amaranth, millet, sorghum, tapioca, polenta, amaranth)
Fruit	Apricots, avocados, persimmons, cherries, figs, lychees, mangoes, apples, blackberries, pears, peaches, nectarines, plums, plums, canned fruit, fruit jams not allowed	Pineapple (1 slice), orange (<130 g), banana (½ banana), clementine (n° 2), strawberries (about n° 10), passion fruit (maracuja), kiwi (<60 g), raspberries (<120 g), mandarin (n° 2), melon (<150 g), blueberries (<70 g), papaya (<80 g), grapefruit (<160 g), grapes (<30 g), strawberry jams, blueberries, raspberries
Vegetables	Garlic, asparagus, beets, broccoli, artichokes, Brussels sprouts, cauliflower, cabbage, chicory, onion, mushrooms, okra, green peppers, leek, shallot, cabbage	Carrots, cucumbers, spring onion (green parts only), ribs, fennel, lettuce and leafy salad, eggplant (<180 g) potatoes, sweet potatoes (½ potato), red peppers (<120 g), tomatoes (less than 200 g), celery (less than 1 stalk), chopped spinach, pumpkin, courgettes (<300 g)
Legumes and nuts	All (chickpeas, beans, lentils, …), pistachios, cashews, soy	Green beans and peas
Desserts	Any prepared with foods to be limited	Any prepared with permitted foods. Dark chocolate
Beverages	Fruit/vegetable juices with a high FODMAP content, beer, barley coffee, spirits, sweet wines	Fruit/vegetable juices with low FODMAP content (coffee (in limited quantities), tea, red and white wine (in limited quantities), herbal teas from permitted foods
Sweeteners	Sorbitol, mannitol, isomalt, maltitol, xylitol, honey, molasses, saccharin, agave or agave syrup, fructose and the syrups that contain it	Small quantities of white or brown sugar, glucose, pure maple syrup, aspartame
Other	Whole or powdered garlic and onion and mix of powdered vegetables (may contain garlic or onion), pickles and spring onions, miso, nuts or vegetable extracts, nuts or meat extracts, ready-made seasonings containing substances to avoid	Vinegar, balsamic vinegar, homemade broth aromatic herbs (e.g., chives, basil, parsley, etc.), bamboo shoots, lemon, mayonnaise, mustard, butter, margarine, canola oil, extra virgin olive oil, spices (pepper, red chili, ginger, cinnamon, etc.), olives, salt, gomasio

**Table 2:** General characteristics of all patients (n. 36), patients with GI manifestation (IBS severity score >75, n. 22), patients adhering to the low-*FODMAP* protocol (n. 11): sex (males, females); age (years); disease form (classical, late, or variant of uncertain significance (VUS)); therapy at the first evaluation (oral, ERT, or no therapy); body mass index (kg/m^2^); adherence to low-*FODMAP* protocol (yes, no, or *in itinere*); IBS severity score; SF-12 score: physical (PCS12) and mental (MCS12).

	Sex	Age	Disease Form	Therapy	Body Mass Index	Low-*FODMAP* Protocol	IBS Severity Score	PCS12	MCS12
All patients (Mean values, n. 36)	36.1% males, 36.9% females	47.6 ± 16.2	63.9% Late, 25% Classical, 11.2% VUS	44.5% ERT, 19.4% Oral, 36.1% none	25.6 ± 6.0	30.5% yes (11,1% *in itinere*), 69.5% no	116.2 ± 97.8	45.6 ± 7.2	51.5 ± 11.2
All patients with GI manifestations (Mean values, n. 22)	27.3% males 72.3% females	47.1 ± 13.2	50% Late, 40.9% Classical, 9.1% VUS	46.1% ERT, 22.7% Oral, 31.2% none	24.6 ± 5.7	50% yes (18.2% *in itinere*), 50% no	181.1 ± 66.8	45.5 ± 6.8	49.8 ± 12.6
All patients adhering to low-*FODMAP* protocol (Mean values, n. 11)	27.3% males 72.7% females	42.3 ± 12.7	36.4% Late, 54.5% Classical, 9% VUS	54.5% ERT, 18.2% Oral, 27.3% none	23.1 ± 5.3	63.6% completed the protocol, 36.3% *in itinere*	195.2 ± 63.5	46.3 ± 8.0	48.7 ± 11.7

**Table 3:** IBS severity score in all FD patients assessed (n. 36): 33.3% presented severe GI manifestations (IBS severity score > 175), 27.7% moderate (75 > IBS severity score > 175), and 38.9% did not show GI symptoms (IBS severity score < 75).

GI Manifestations	N. of Patients	Mean IBS Severity Scores ± SD
**No**	14 (38.9%)	13.4 ± 14.3 (0–45)
**Moderate**	10 (27.7%)	120.6 ± 31.8 (83–170)
**Severe**	12 (33.3%)	231.5 ± 40.1 (190–290)

## Supplementary Materials

The following supporting information can be downloaded at: https://www.mdpi.com/article/10.3390/nu15030658/s1, General characteristics of the FD patients in detail for each subject. Figure S1: IBS severity score in all subjects (n. 36): severe GI manifestations in 33.3% of patients, moderate in 27.7%, no GI symptoms in 38.9% of subjects; Table S1: Patients’ general characteristics: sex (35.1% males, 64.9% females); age (years); genetic (gene mutation); disease form (classical, late, or variant of uncertain significance (VUS)); therapy at the first evaluation (oral, ERT, or no therapy); body mass index (kg/m^2^); adherence to low-*FODMAP* protocol (yes, no or *in itinere*); IBS severity score; SF-12 score: physical (PCS12) and mental (MCS12).

## References

[1] Meikle P.J., Hopwood J.J., Clague A.E., Carey W.F. (1999). Prevalence of lysosomal storage disorders. JAMA.

[2] Hopkins P.V., Klug T., Vermette L., Raburn-Miller J., Kiesling J., Rogers S. (2018). Incidence of 4 Lysosomal Storage Disorders From 4 Years of Newborn Screening. JAMA Pediatr..

[3] Zarate Y.A., Hopkin R.J. (2008). Fabry’s disease. Lancet.

[4] El-Abassi R., Singhal D., England J.D. (2014). Fabry’s disease. J. Neurol. Sci..

[5] Radulescu D., Crisan D., Militaru V., Buzdugan E., Stoicescu L., Grosu A., Vlad C., Grapa C., Radulescu M.L. (2022). Gastrointestinal Manifestations and Treatment Options in Fabry Disease Patients. A Systematic Review. J. Gastrointestin. Liver Dis..

[6] Mehta A., Clarke J.T.R., Giugliani R., Elliott P., Linhart A., Beck M., Sunder-Plassmann G. (2009). Natural course of Fabry disease: Changing pattern of causes of death in FOS-Fabry Outcome Survey. J. Med. Genet..

[7] Lenders M., Brand E. (2022). Fabry disease–a multisystemic disease with gastrointestinal manifestations. Gut Microbes.

[8] Hoffmann B., Schwarz M., Mehta A., Keshav S. (2007). Gastrointestinal symptoms in 342 patients with Fabry disease: Prevalence and response to enzyme replacement therapy. Clin. Gastroenterol. Hepatol..

[9] Zar-Kessler C., Karaa A., Sims K.B., Clarke V., Kuo B. (2016). Understanding the gastrointestinal manifestations of Fabry disease: Promoting prompt diagnosis. Therap. Adv. Gastroenterol..

[10] Spada M., Pagliardini S., Yasuda M., Tukel T., Thiagarajan G., Sakuraba H., Ponzone A., Desnick R.J. (2006). High incidence of later-onset fabry disease revealed by newborn screening. Am. J. Hum. Genet..

[11] Ortiz A., Germain D.P., Desnick R.J., Politei J., Mauer M., Burlina A., Eng C., Hopkin R.J., Laney D., Linhart A. (2018). Fabry disease revisited: Management and treatment recommendations for adult patients. Mol. Genet. Metab..

[12] Biegstraaten M., Arngrímsson R., Barbey F., Boks L., Cecchi F., Deegan P.B., Feldt-Rasmussen U., Geberhiwot T., Germain D.P., Hendriksz C. (2015). Recommendations for initiation and cessation of enzyme replacement therapy in patients with Fabry disease: The European Fabry Working Group consensus document. Orphanet J. Rare Dis..

[13] Friis H., Bodé S., Rumessen J.J., Gudmand-Høyer E. (1991). Effect of simethicone on lactulose-induced H2 production and gastrointestinal symptoms. Digestion.

[14] Argoff C.E., Barton N.W., Brady R.O., Ziessman H.A. (1998). Gastrointestinal symptoms and delayed gastric emptying in Fabry’s disease: Response to metoclopramide. Nucl. Med. Commun..

[15] Bassotti G., Usai-Satta P., Bellini M. (2018). Linaclotide for the treatment of chronic constipation. Expert Opin. Pharmacother..

[16] Page J.G., Dirnberger G.M. (1981). Treatment of the irritable bowel syndrome with Bentyl (dicyclomine hydrochloride). J. Clin. Gastroenterol..

[17] Francini-Pesenti F., Ravarotto V., Bertoldi G., Spinella P., Calò L.A. (2020). Could nutritional therapy take us further in our approaches to Fabry disease?. Nutrition.

[18] Liu J., Chey W.D., Haller E., Eswaran S. (2020). Low-FODMAP Diet for Irritable Bowel Syndrome: What We Know and What We Have Yet to Learn. Annu. Rev. Med..

[19] Murray K., Wilkinson-Smith V., Hoad C., Costigan C., Cox E., Lam C., Marciani L., Gowland P., Spiller R.C. (2014). Differential effects of FODMAPs (fermentable oligo-, di-, mono-saccharides and polyols) on small and large intestinal contents in healthy subjects shown by MRI. Am. J. Gastroenterol..

[20] Chey W.D., Hashash J.G., Manning L., Chang L. (2022). AGA Clinical Practice Update on the Role of Diet in Irritable Bowel Syndrome: Expert Review. Gastroenterology.

[21] Carubbi F., Barbato A., Burlina A.B., Francini F., Mignani R., Pegoraro E., Landini L., De Danieli G., Bruni S., Strazzullo P. (2021). Nutrition in adult patients with selected lysosomal storage diseases. Nutr. Metab. Cardiovasc. Dis..

[22] Hill C., Guarner F., Reid G., Gibson G.R., Merenstein D.J., Pot B., Morelli L., Canani R.B., Flint H.J., Salminen S. (2014). Expert consensus document. The International Scientific Association for Probiotics and Prebiotics consensus statement on the scope and appropriate use of the term probiotic. Nat. Rev. Gastroenterol. Hepatol..

[23] Marco M.L., Sanders M.E., Gänzle M., Arrieta M.C., Cotter P.D., De Vuyst L., Hill C., Holzapfel W., Lebeer S., Merenstein D. (2021). The International Scientific Association for Probiotics and Prebiotics (ISAPP) consensus statement on fermented foods. Nat. Rev. Gastroenterol. Hepatol..

[24] Hilz M.J., Arbustini E., Dagna L., Gasbarrini A., Goizet C., Lacombe D., Liguori R., Manna R., Politei J., Spada M. (2018). Non-specific gastrointestinal features: Could it be Fabry disease?. Dig. Liver Dis..

[25] Cozma-Petrut A., Loghin F., Miere D., Dumitrascu D.L. (2017). Diet in irritable bowel syndrome: What to recommend, not what to forbid to patients!. World J. Gastroenterol..

[26] Staudacher H.M., Whelan K. (2017). The low FODMAP diet: Recent advances in understanding its mechanisms and efficacy in IBS. Gut.

[27] Ware J.E., Kosinski M., Keller S.D. (1996). A 12-Item Short-Form Health Survey: Construction of scales and preliminary tests of reliability and validity. Med. Care.

[28] Wiklund I.K., Fullerton S., Hawkey C.J., Jones R.H., Longstreth G.F., Mayer E.A., Peacock R.A., Wilson I.K., Naesdal J. (2003). An irritable bowel syndrome-specific symptom questionnaire: Development and validation. Scand. J. Gastroenterol..

[29] Francis C.Y., Morris J., Whorwell P.J. (1997). The irritable bowel severity scoring system: A simple method of monitoring irritable bowel syndrome and its progress. Aliment. Pharmacol. Ther..

[30] Battaglia Y., Fiorini F., Azzini C., Esposito P., De vito A., Granata A., Storari A., Mignani R. (2021). Deficiency in the Screening Process of Fabry Disease: Analysis of Chronic Kidney Patients Not on Dialysis. Front. Med..

[31] Svarstad E., Marti H.P. (2020). The Changing Landscape of Fabry Disease. Clin. J. Am. Soc. Nephrol..

[32] Jahan S., Sarathchandran S., Akhter S., Goldblatt J., Stark S., Crawford D., Mallett A., Thomas M. (2020). Prevalence of Fabry disease in dialysis patients: Western Australia Fabry disease screening study-the FoRWARD study. Orphanet J. Rare Dis..

[33] Politei J., Thurberg B.L., Wallace E., Warnock D., Serebrinsky G., Durand C., Schenone A.B. (2016). Gastrointestinal involvement in Fabry disease. So important, yet often neglected. Clin. Genet..

[34] Jack C.I.A., Morris A.I., Nasmyth D.G., Carroll N. (1991). Colonic involvement in Fabry’s disease. Postgrad. Med. J..

[35] MacDermot K.D., Holmes A., Miners A.H. (2001). Anderson-Fabry disease: Clinical manifestations and impact of disease in a cohort of 98 hemizygous males. J. Med. Genet..

[36] Bellini M., Tonarelli S., Nagy A.G., Pancetti A., Costa F., Ricchiuti A., de Bortoli N., Mosca M., Marchi S., Rossi A. (2020). Low FODMAP Diet: Evidence, Doubts, and Hopes. Nutrients.

