# Supplementary material for: Gastrointestinal Manifestations and Low-FODMAP Protocol in a Cohort of Fabry Disease Adult Patients
Source: Nutrients. 2023 Jan 28;15(3):658. doi: 10.3390/nu15030658 (PMC9920936; doi:10.3390/nu15030658)
Supplement: Supplementary file 1 [file nutrients-15-00658-s001.zip › nutrients-2166831-supplementary.pdf]

nutrients

MDPI

Gastrointestinal Manifestations and Low-*FODMAP* Protocol in a Cohort of Fabry Disease Adult Patients

Supplementary Materials: General characteristics of the FD patients in detail for each subject.

Table S1. Patients' general characteristics: sex (35,1% males, 64,9% females); age (years); genetic (gene mutation); disease form (classical, late or variant of uncertain significance (VUS)); therapy at the first evaluation (oral, ERT or no therapy); body mass index (kg/m2); adherence to Low FODMAPs protocol (yes, no or *in itinere*); IBS severity score; SF-12 score: physical (PCS12) and mental (MCS12).

Patient	Sex	Age	Genetic	Disease form	Therapy	Body Mass Index	Low Fodmaps protocol	IBS severity score	PCS12	MCS12
1	M	48,9	GLA	Late	Oral	29,0	no	190	46,0	28,2
2	F	43,5	GLA	Late	ERT	20,3	yes	200	52,8	54,5
3	M	16,8	GLA	Late	No therapy	26,1	no	2	40,8	62,7
4	M	18,9	GLA	Late	No therapy	23,7	no	2	41,8	63,7
5	F	35,6	GLA	Late	Oral	24,9	no	85	53,2	43,2
6	M	56,1	GLA	Classic	ERT	21,0	yes	148	32,6	53,6
7	F	31,1	GLA	Late	No therapy	20,2	*in itinere*	276	46,8	68,7
8	F	61,0	GLA	Late	No therapy	19,8	no	276	47,8	69,7
9	M	46,5	GLA	Classic	ERT	20,3	no	170	39,8	61,7
10	F	32,4	GLA	Classic	ERT	20,8	yes	270	40,6	37,7
11	F	26,4	GLA	Classic	ERT	19,2	no	145	52,2	36,6
12	F	23,5	GLA	Classic	ERT	23,9	yes	245	45,6	30,9
13	M	62,5	GLA	Classic	ERT	23,7	no	290	42,8	64,7
14	F	59,8	GLA	Late	No therapy	37,7	no	183	33,6	59,1
15	F	56,8	GLA	Late	No therapy	44,5	no	33	54,1	42,9
16	F	19,2	GLA	VUS	No therapy	29,3	no	20	52,7	50,7
17	F	59,8	GLA	Classic	No therapy	20,4	*in itinere*	193	55,1	53,3
18	M	30,3	GLA	Classic	No therapy	18,4	*in itinere*	110	56,6	53,7
19	F	49,5	GLA	Late	No therapy	25,2	no	130	43,8	65,7
20	M	32,3	GLA	VUS	No therapy	23,3	no	5	45,8	67,7
21	F	47,4	GLA	VUS	No therapy	35,2	no	83	43,6	49,7
22	M	50,2	GLA	Late	Oral	22,6	no	20	60,0	47,7
23	M	49,1	GLA	Classic	ERT	23,0	yes	220	55,0	35,9
24	F	65,2	GLA	Late	No therapy	24,8	no	5	36,2	49,3
25	F	57,4	GLA	Late	Oral	24,9	no	95	48,8	38,7
26	M	60,2	GLA	Late	ERT	33,8	no	2	44,8	66,7
27	M	58,9	GLA	Late	Oral	31,1	no	25	52,5	46,8
28	F	51,0	GLA	Late	Oral	29,9	yes	245	46,7	38,1

29	F	56,2	GLA	VUS	Oral	36,0	yes	150	41,3	48,9
30	F	75,1	GLA	Late	ERT	27,4	no	190	41,1	42,3
31	F	82,0	GLA	Late	ERT	23,8	no	5	30,5	61,3
32	F	48,3	GLA	Late	ERT	18,1	no	5	41,9	52,1
33	F	52,0	GLA	Late	ERT	23,6	no	25	53,6	44,8
34	M	75,6	GLA	Late	ERT	24,2	no	5	41,9	52,1
35	F	48,3	GLA	Late	ERT	31,4	no	45	43,3	49,5
36	F	32,8	GLA	Late	ERT	20,1	*in itinere*	90	36,5	60,6
**Mean values**		**47,6**	**GLA**	**63.9% Late, 25% Classical, 11.2% VUS**	**44,5% ERT, 19,4% Oral, 36,1% none**	**25,6**	**19,4% yes, 11,1& *in itinere*, 68,7% no**	**116,2**	**45,6**	**51,5**

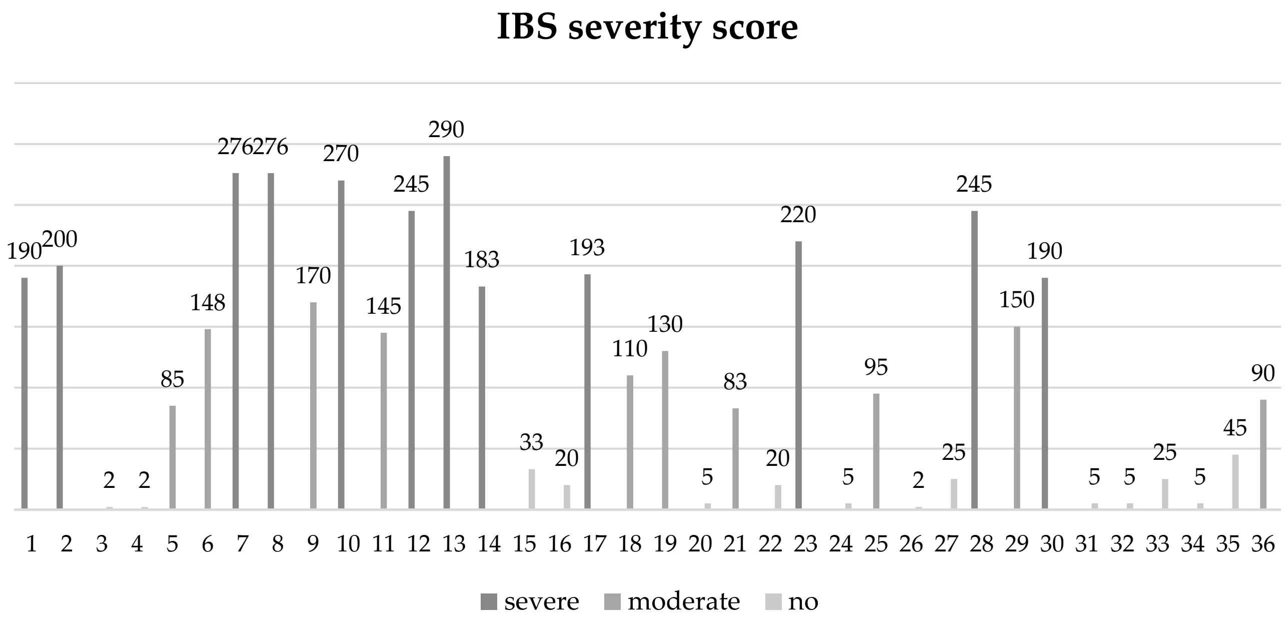

Figure S1. IBS severity score in all subjects (n. 36): severe GI manifestations in 33,3% of patients, moderate in 27,7%, no GI symptoms in 38,9% of subjects.

Disclaimer/Publisher's Note: The statements, opinions and data contained in all publications are solely those of the individual author(s) and contributor(s) and not of MDPI and/or the editor(s). MDPI and/or the editor(s) disclaim responsibility for any injury to people or property resulting from any ideas, methods, instructions or products referred to in the content.
